# Prophylactic Central Neck Dissection to Improve Disease-Free Survival in Pediatric Papillary Thyroid Cancer

**DOI:** 10.3389/fonc.2022.935294

**Published:** 2022-07-28

**Authors:** Duy Quoc Ngo, Duong The Le, Quang Le

**Affiliations:** ^1^ Department of Head and Neck Surgery, Vietnam National Cancer Hospital, Hanoi, Vietnam; ^2^ Department of Oncology, Hanoi Medical University, Hanoi, Vietnam

**Keywords:** prophylactic central neck dissection (CND), pediatric thyroid cancer, papillary thyroid cancer (PTC), pediatric thyroid surgery, pediatric thyroid carcinoma

## Abstract

**Background:**

Pediatric PTC is a rare disease. Although, the prognosis is excellent and the mortality rate is low, the role of prophylactic central neck dissection is still the subject of debate. The aim was to evaluate both the role and safety of prophylactic central neck dissection (CND) in managing pediatric papillary thyroid cancer (PTC), especially in respect of disease-free survival (DFS).

**Patients and Methods:**

In this retrospective study, we collected 54 pediatric cN0 PTC patients (≤ 18 years of age) who were treated from January 2014 to January 2021 at a high-volume thyroid surgery center. Patients were divided into two groups based on the status of prophylactic CND. We analyzed the factors related to the clinicopathological features and recurrence of PTC in children.

**Results:**

Of the 54 cN0 patients, 35 underwent prophylactic CND and 19 patients did not undergo prophylactic CND. The two groups were similar in clinical and pathologic features, such as age, gender, tumor size, multifocal status, and follow-up time. The average DFS was 84.4 ± 2.7 months. Log-rank tests on Kaplan-Meier curves revealed that age, gender, tumor size, multifocality, and extrathyroid extension did not relate to DFS time. Furthermore, DFS time was not affected by the extent of thyroidectomy (p=0.07) or RAI treatment (p=0.21). Prophylactic CND was found to increase DFS time for pediatric patients with cN0 PTC (p = 0.003). There was no statistically significant difference in complications such as transient hypocalcemia (p=0.15) and transient recurrent laryngeal nerve injury (p=0.37) between the prophylactic CND group and the no-prophylactic CND group.

**Conclusion:**

Prophylactic CND was found to be associated with increased DFS and not with increased rates of complications after surgery.

## Introduction

Most pediatric thyroid cancers are differentiated thyroid cancer (DTC), with papillary thyroid cancer (PTC) comprising 90% of DTC. According to the American Thyroid Association, pediatric thyroid cancer (PTC) is a rare disease but with incidence increasing by approximately 3% annually (1). PTC accounts for approximately 1.5 percent of all malignancies in the under-18 age group, with an age-adjusted incidence of 4.8 to 5.9 per 1,000,000 people (2).

Compared to adults, thyroid cancer in children more commonly presents at an advanced stage, with increased extrathyroidal extension, cervical lymph node metastasis, and distant metastasis (3, 4). However, the prognosis is excellent and the mortality rate is low, although optimal treatment strategies for children with PTC remain controversial (1, 5). Specifically, the role of prophylactic central neck dissection (CND) is still the subject of debate. Several studies have shown that prophylactic CND improves disease-free survival (6–8). On the other hand, prophylactic CND is associated with increased complications, such as hypoparathyroidism and recurrent laryngeal nerve injury, that negatively affect quality of life, especially in children (1). Thus, we aim to assess the role of prophylactic CND in the management of PTC in children, as well as the potential risks after surgery.

## Patients and Methods

### Patients

In this retrospective study, we collected 54 pediatric papillary thyroid cancer patients with cN0 stage cancer who were treated from January 2014 to January 2021 at the National Cancer Hospital (a high-volume thyroid surgery center in Vietnam with 1500-1800 thyroid surgeries in adults and 20-30 operations for thyroid cancer in children annually). The study was conducted after obtaining informed consent from patients and their parents in compliance with ethical guidelines. This study was approved by the institution’s ethics committee and research board.

Patient selection criteria: pediatric PTC patients (≤ 18 years of age) who had no neck lymph node metastases (cN0), as shown by both ultrasonography and clinical examination; a pathology finding after surgery of PTC: total thyroidectomy and prophylactic CND on children with stage cT3-4N0M0 PTC or bilateral tumors; total thyroidectomy or lobectomy plus isthmusectomy with or without prophylactic central cervical lymph node dissection on children with unilateral PTC at stage cT1-2N0M0.

Patient exclusion criteria: patients with distant metastases, recurrence, or a history of previous thyroid surgery.

### Objectives

The aim of this study was to evaluate the role of prophylactic CND in managing pediatric PTC, especially in respect of recurrence of the disease. We also investigated the safety of prophylactic CND, specifically, postoperative complications related to the treatment.

### Treatment

Before surgery: initial evaluation of children with PTC included a detailed clinical examination, neck ultrasound, laryngoscopy to examine the function of the vocal cords, blood tests (TSH, FT4) to evaluate thyroid gland activity, and serum Tg and anti-Tg. All the children underwent fine-needle aspiration biopsy which revealed PTC. A neck ultrasound scan was performed by two experienced radiologists to confirm that there were no neck lymph node metastases.

Surgery: all the children were operated on by the same high-volume surgical team.

Post-operative treatment: radioactive iodine (RAI) therapy was used, based on the American Thyroid Association’s treatment guidelines and the results of Multidisciplinary Thyroid Meeting consultations at our hospital.^1^ The dose of RAI therapy for children is 1 mCi/kg body weight.

### Follow-up

Transient hypoparathyroidism was defined as a serum calcium concentration below 8 mg/dL or when patients had symptomatic hypocalcemia less than 6 months after surgery. Patients who had hypocalcemia after more than 6 months were classified as having permanent hypoparathyroidism. Transient recurrent laryngeal nerve injury was confirmed by a laryngoscopic diagnosis after surgery and when both vocal cord functions recovered fully within 6 months of surgery. Children were classified as having permanent recurrent laryngeal nerve injury if these findings persisted beyond 6 months.

Clinical examination, neck ultrasound, and biochemical assessment (serum Tg and anti-Tg) were performed on the children every three months for the first two years, every six months up to five years, and then once a year. After RAI treatment, TSH-stimulated Tg and whole-body iodine scans were used to evaluate the effectiveness of the therapy.

The post-treatment response was evaluated according to the American Thyroid Association guidelines for children with PTC published in 2016 (1). Disease-free survival (DFS) is defined as the time interval from initial therapy to detection of recurrent PTC. Local recurrence is defined as evidence of PTC in the neck region detected by imaging (CT, MRI, PET/CT, etc.) and confirmed by fine-needle aspiration biopsy. Distant metastasis is defined as PTC identified outside the neck by an imaging study (CT, PET, whole-body iodine scan, etc.) or biopsy. Serum shTSH-stimulated Tg or unstimulated Tg and anti-Tg were assessed to assist in diagnosing distant metastatic disease.

### Statistical Analysis

Statistical analysis was performed using SPSS (Statistical Package for Social Sciences) version 25.0 (IBM Corp. IMB SPSS Statistics, Armonk, NY). Results of continuous variables are expressed as mean ± SD (min, max) and for categorical variables as absolute numbers or percentages. When comparing categorical data, the χ^2^ test or, if deemed appropriate, Fisher’s exact test were used, while the T-test was used to compare continuous data. Factors associated with recurrent disease were examined by univariate Cox analysis. A p-value of less than 0.05 was considered significant.

## Results

Of the 54 patients in our study, the majority were female (accounting for 77.8%) and aged 15 years or older (accounting for 79.6%). None of the patients had any family history of cancer or prior neck radiation therapy. The average tumor size was 15.9 ± 7.8 mm (4 - 35 mm). Most tumors were over 10 mm (72.2%) and TIRADS 5 (75.9%). Only five patients had multifocal cancer, and seven had an extrathyroid extension. Stage T1a, T1b, T2, T3b accounted for 27.8%, 33.3%, 25.9%, and 13.0%, respectively. Of the 54 patients, 38.9% underwent lobectomy plus isthmusectomy and 61.1% underwent total thyroidectomy **Table 1**.

**Table 1:** Clinicopathological features of cN0 papillary thyroid cancer in children.

Features	Distributions (n=54), %
**Gender**, n (%)	
Female	42 (77.8)
Male	12 (22.2)
Age, mean (SD)^a^	16.0 ± 2.5
< 15	11 (20.4)
≥ 15	43 (79.6)
**History**, n (%)	
Previous radiation exposure	0 (0)
Normal	54 (100)
**Tumor size (mm)**	
Mean ± SD: 15.9 ± 7.8 (4 – 35)	
≤ 10 mm	15 (27.8)
> 10 mm	39 (72.2)
**Location**, n (%)	
Right lobe	28 (51.9)
Left lobe	23 (42.6)
Both lobes	3 (5.5)
**Multifocality**, n (%)	
Uni focus	49 (90.7)
Multi foci	5 (9.3)
**TIRADS**, n (%)	
TIRADS 4	13 (24.1)
TIRADS 5	41(75.9)
**Extrathyroidal extension**, n (%)	
Yes	7 (13.0)
No	47 (87.0)
**Primary tumor (T)**, n (%)	
T1a	15 (27.8)
T1b	18 (33.3)
T2	14 (25.9)
T3b	7 (13.0)
**Regional lymph nodes (N)**, n (%)	
N0	27 (50.0)
N1a	27 (50.0)
**Distant metastasis (M)**, n (%)	
Yes (Lung)	0 (0)
No	54 (100)
**Type of thyroidectomy**, n (%)	
Lobectomy	21 (38.9)
Total thyroidectomy	33 (61.1)
**Radioactive Iodine,** n (%)	
Yes	28 (51.9)
No	26 (48.1)
**Prophylactic central neck dissection**, n (%)	
Yes	35 (64.8)
No	19 (35.2)

a SD, standard deviation.

Patients were divided into two groups based on the status of prophylactic CND. Of the 54 cN0 patients, 35 underwent prophylactic CND (prophylactic CND group) (accounting for 64.8%) and 19 patients did not undergo prophylactic CND (no-prophylactic CND group, accounting for 35.2%). The two groups were similar in clinical and pathologic features, such as age, gender, tumor size, multifocal status, and follow-up time. However, extrathyroidal extension, total thyroidectomy, RAI treatment, and central lymph node metastasis were significantly more frequent in the prophylactic group (**Table 2**). RAI therapy was indicated significantly more often in the prophylactic group (68.6% vs 21.1%, p=0.001). Furthermore, the patients who underwent prophylactic CND had significantly lower rates of recurrence than the no-prophylactic CND group at the same follow-up time (2.9% vs 31.6%, p < 0.006).

**Table 2:** Clinicopathological features according to prophylactic central neck dissection.

Variables	Prophylactic CND (n=35)	No prophylactic CND (n=19)	P-value
**Age (years)**			
< 15	7 (20%)	4 (21.1%)	p = 0.927
≥ 15	28 (80%)	15 (78.9%)	
**Gender**, n (%)			
Female	26 (74.3%)	16 (84.2%)	p = 0.402
Male	9 (25.7%)	3 (15.8%)	
**Tumor size**, n (%)			
≤ 10 mm	7 (20%)	2 (10.5%)	p = 0.372
> 10 mm	28 (80%)	17 (89.5%)	
**Mutifocality**, n (%)			
Uni focus	32 (91.4%)	17 (89.5%)	p = 0.813
Multi foci	3 (8.6%)	2 (10.5%)	
**Extrathyroidal extension**, n (%)			
Yes	7 (20%)	0 (0%)	p = 0.037
No	28 (80%)	19 (100%)	
**Type of thyroidectomy**, n (%)			
Lobectomy	5 (14.3%)	16 (84.2%)	p < 0.001
Total thyroidectomy	30 (85.7%)	3 (15.8%)	
**Regional lymph nodes (N)**, n (%)			
N0	8 (22.9%)	19 (100%)	p < 0.001
N1a	27 (77.1%)	0 (0%)	
**Radioactive Iodine,** n (%)			
Yes	24 (68.6%)	4 (21.1%)	p = 0.001
No	11 (31.4%)	15 (78.9%)	
**Follow-up, months**	71.7 ± 10.6	71.3 ± 15.3	p = 0.905
**Recurrent disease**, n (%)			
Yes	1 (2.9%)	6 (31.6%)	p = 0.006
No	34 (97.1%)	13 (68.4%)	

CND, central neck dissection.

### Recurrence

**Figure 1:**
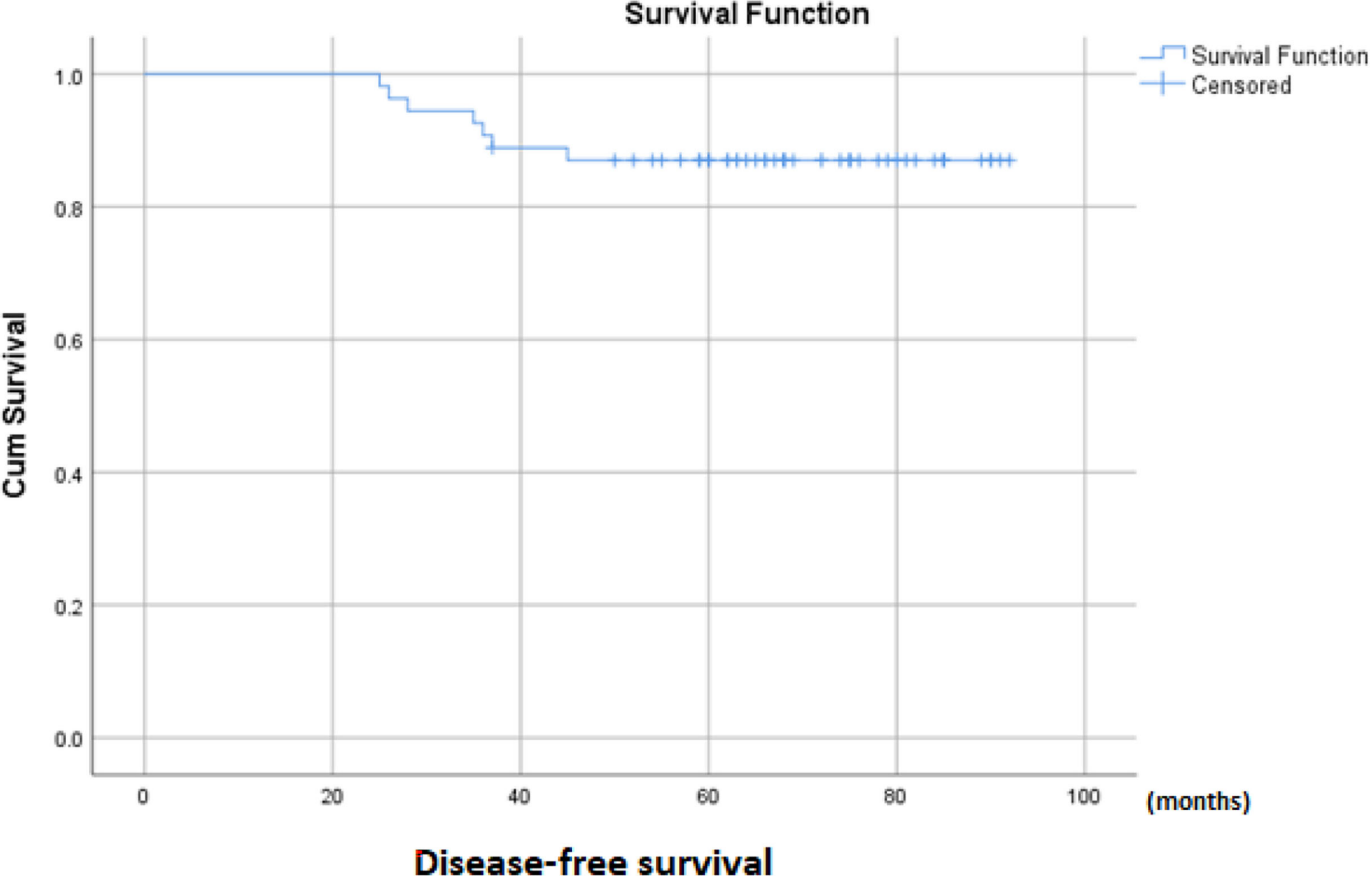
Kaplan–Meier curves estimating disease-free survival of pediatric cN0 papillary thyroid cancer.

**Figure 2:**
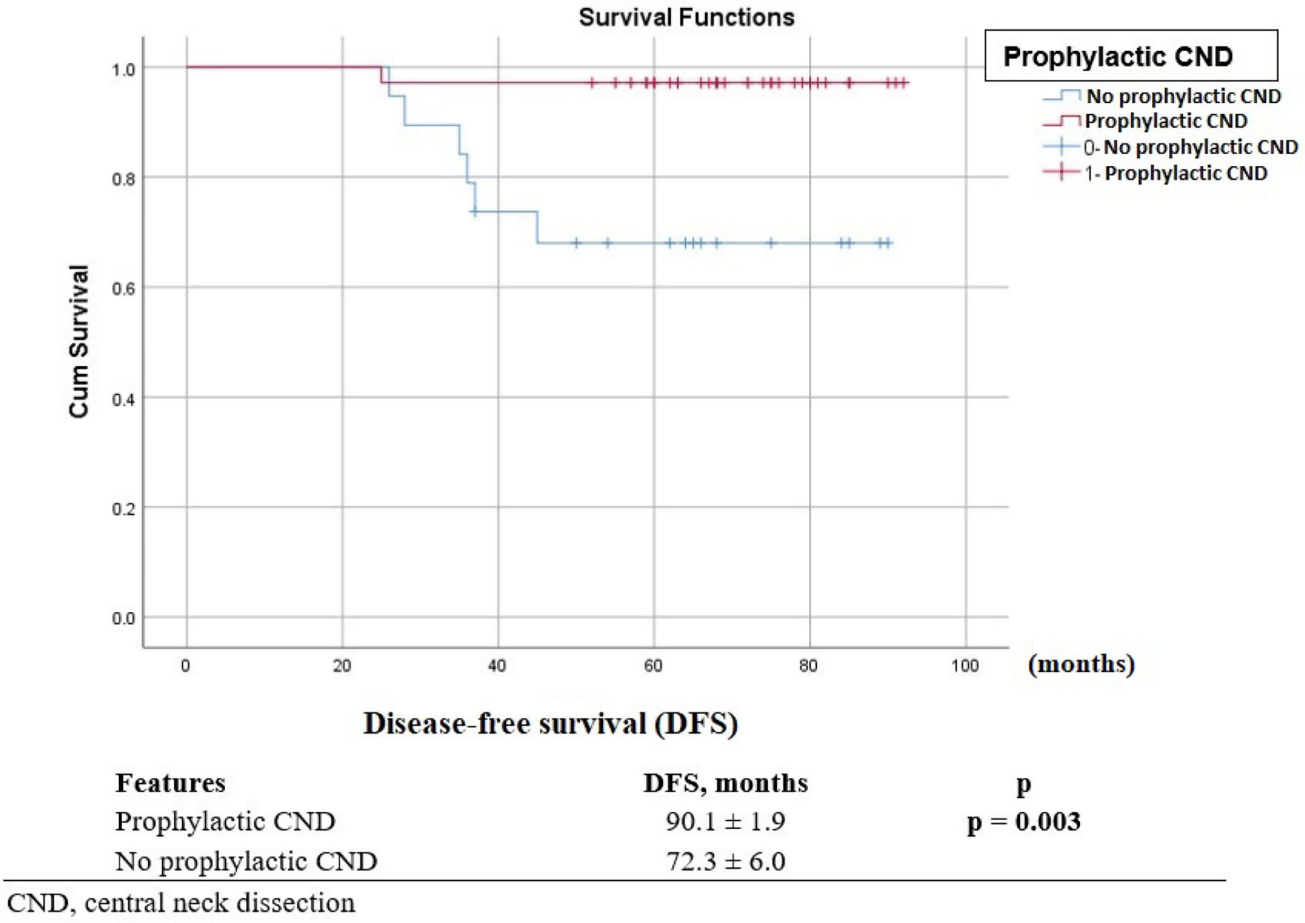
Kaplan–Meier curves estimating disease-free survival according to surgical procedure.

There was no significant difference in follow-up time between the two groups (71.7 ± 10.6 months vs 71.3 ± 15.3 months, p = 0.905). Of the 54 patients, seven patients had locoregional recurrence within the follow-up time. Of these, all had central lymph node metastasis. In addition, only two patients were found to have lateral compartment as well as central compartment recurrence. The average DFS was 84.4 ± 2.7 months **Figure 1**. Log-rank tests on Kaplan-Meier curves revealed that age, gender, tumor size, multifocality, and extrathyroid extension did not relate to DFS time. Univariate analysis showed that DFS time was not affected by the extent of thyroidectomy or RAI treatment (**Table 3**). Both univariate analysis and multivariate analysis show that prophylactic CND increased DFS time for pediatric patients with cN0 PTC (**Tables 3**, **4**). Kaplan Meier curves show that patients who underwent prophylactic CND had a significantly better DFS than those who did not (p = 0.03; **Figure 2**).

**Table 3:** Univariate analysis of risk factors for recurrence in pediatric patients.

Variables	P value
Age (years)	p = 0.596
Gender	p = 0.699
Tumor size ≤ 10 mm	p = 0.219
Mutifocality	p = 0.382
Extrathyroidal extension	p = 0.858
Type of thyroidectomy	p = 0.069
Radioactive Iodine	p = 0.214
Prophylactic central neck dissection	p = 0.003

**Table 4:** Multivariate analysis of risk factors for recurrence in pediatric patients.

Variables	P value
Type of thyroidectomy	p = 0.693
Radioactive Iodine	p = 0.815
Prophylactic central neck dissection	p = 0.035

### Safety of Prophylactic CND

To determine the effectiveness of prophylactic CND, associated complications were analyzed. Hypoparathyroidism and recurrent laryngeal nerve injury are two major consequences of thyroid surgery that have a negative impact on a child’s quality of life. There was no statistically significant difference in complications between the prophylactic CND group and the no-prophylactic CND group in terms of temporary hypoparathyroidism (20% vs 5.3%, p = 0.145), temporary recurrent laryngeal nerve injury (20% vs 10.5%, p = 0.372), postoperative bleeding, permanent hypoparathyroidism, or permanent recurrent laryngeal nerve injury (**Table 5**).

**Table 5:** Postoperative complications for patients with cN0 pediatric thyroid carcinoma.

Postoperative complications	Prophylactic CND n, (%)	No prophylactic CND n, %	p, Univariable Model OR (95% CI)
Transient hypocalcemia	7 (20%)	1 (5.3%)	p = 0.145
Permanent hypocalcemia	0	0	N/A
Transient RLN injury	7 (20%)	2 (10.5%)	p = 0.372
Permanent RLN injury	0	0	N/A
Postoperative bleeding	0	0	N/A

CND, central neck dissection; RLN, recurrent laryngeal nerve.

## Discussion

### The rate of Central Lymph Node Metastasis in Pediatric Patients With cN0 PTC Undergoing Prophylactic CND

In our study, 35 pediatric patients with cN0 PTC underwent prophylactic CND. Of these, 27 patients were found to have central lymph node metastasis, accounting for 77.1%. The rate of central lymph node metastasis in children with the cN0 disease has been shown to be higher than in other age groups (9). In a meta-analysis of 17 studies, Hughes et al. found the rate in other age groups to be approximately 50% (10). In a meta-analysis of 31 studies involving 37,355 patients with cN0 PTC in various age groups, the rate of central lymph node metastasis was reported as 26.4% (11). Thus, the rate of central lymph node metastasis in cN0 pediatric patients is extremely high, which may suggest that prophylactic CND is beneficial in the treatment of PTC in children.

#### Disease-Free Survival and Prophylactic CND

Despite more widespread disease at discovery compared to adults, children with thyroid cancer have higher survival rates, even in those with distant metastasis or recurrent disease. In follow-up, seven patients (13.0%) experienced recurrence in cervical lymph nodes but had no distant metastases. Total thyroidectomy (if not already removed), cervical lymphadenectomy, and iodine 131 therapy were performed on these patients. Kaplan Meier curves show that the DFS time was 84.4 ± 2.7 months, and the 3-year and 5-year DFS rates were 89% and 87%, respectively..

Most studies report recurrence rates of 20% to 40% 10 years after initial treatment of the disease (5, 12–15). Mechteld et al. found that 72 children with DTC (<18 years) who were treated at a single institution between 2003 and 2018 had a median DFS time of 36.7 months, with a 1-year recurrence-free survival (RFS) rate of 93% and a 5-year RFS of 87% (16). A national multicenter retrospective review of 250 pediatric patients treated for PTC in Italy with an average follow-up of 5.8 years found that the rate of recurrent disease was 12% (30/250). Of the 30 recurrent patients, 12 experienced recurrence in the first year and eight in the second year. The main location of recurrence was the cervical lymph nodes (accounting for 56.7%), followed by lung metastases (23.3%), and the thyroid bed (20%) (14).

The utility of prophylactic CND in children with PTC is uncertain. The increased incidence of central cervical metastasis in pediatric patients suggests that prophylactic CND should be considered at the time of initial surgery for children with PTC. On the other hand, lymph node dissection increases complications that negatively affect quality of life, especially in children. Thus, a balance has to be found between the advantages and disadvantages of prophylactic CND in the treatment of PTC in children.

Age, gender, tumor size, multifocality, extrathyroid extension, extent of thyroidectomy and RAI treatment were found not to be related to RFS rates. Importantly, pediatric patients who underwent prophylactic CND show significantly better RFS rates. Moreover, prophylactic CND allows for more accurate staging of the tumor, ensuring better assessment of the N status. The rate of central lymph node metastasis was significantly higher in the prophylactic CND group than in the other group, but more patients underwent RAI therapy after surgery in the prophylactic CND group (p=0.001). However, DFS time was not affected by RAI treatment.

#### Complications

Multiple studies have established that complication rates after pediatric thyroidectomy are higher than after similar surgery in adult patients. Because pediatric patients generally have an excellent life expectancy, even in the setting of advanced disease, they are at risk of enduring the long-term effects of these complications, particularly those involving calcium metabolism and airway compromise. According to current guidelines for the treatment of thyroid cancer, pediatric thyroid surgery should ideally be performed by high-volume surgeons. In our hospital, a high-volume thyroid surgery center in Vietnam with 1500-1800 thyroid surgeries in adults and 20-30 operations for thyroid cancer in children annually, the rate of postoperative complications is low. The most common complications after thyroidectomy are hypoparathyroidism and vocal fold paralysis. Our results show that the rate of temporary hypoparathyroidism is 14.8%, and there were no cases of permanent hypoparathyroidism. Also, 16.7% of the cases exhibited temporary recurrent laryngeal nerve damage, but there were no cases of permanent recurrent laryngeal nerve damage. In our high-volume center, the proportion of complications is lower than in other surgery centers. Mechteld et al. found a rate of hypocalcemia of 37.5%, with long-term hypoparathyroidism persisting in 18 patients (25.0%) (16). In a 21-year study of 184 PTC patients, transient and permanent hypoparathyroidism occurred in 33.1% and 3.3% of cases, respectively (17).

More importantly, there is no statistically significant difference in complications between the prophylactic CND group and the no-prophylactic CND group in terms of temporary hypoparathyroidism (20% vs 5.3%, p = 0.145), temporary recurrent laryngeal nerve injury (20% vs 10.5%, p = 0.372), postoperative bleeding, permanent hypoparathyroidism, or permanent recurrent laryngeal nerve injury.

This study had some limitations. First, this was a single center, retrospective study. Second, the number of pediatric patients with cN0 was small. Patients were not randomized, and groups were not equal in risk, as PTC is a rare disease that more commonly presents at an advanced stage with cervical lymph node metastasis. Third, the prognosis is excellent in children and recurrences can appear up to 10 years after treatment; thus, additional long-term follow-up would be needed to confirm these findings. These limitations need to be addressed in the future. Nonetheless, this was the first article reporting the role of prophylactic CND in the management of cN0 PTC in children, to the best of our knowledge. All patients had undergone high-volume thyroid surgery, and complications can be minimized to improve the treatment of thyroid cancer, especially in pediatric patients.

## Conclusion

In summary, prophylactic CND was found to be associated with increased DFS but not with increased rates of complications after surgery. To the best of our knowledge, this is the first article reporting the role of prophylactic CND in the management of cN0 PTC in children. However, larger sample size studies with long-term follow-up are needed to explore further the role of prophylactic CND in treating pediatric patients with PTC.

## Data Availability Statement

The datasets presented in this article are not readily available because due to the nature of this research, participants of this study did not agree for their data to be shared publicly, so supporting data is not available. Requests to access the datasets should be directed to duyyhn@gmail.com.

## Ethics Statement

The studies involving human participants were reviewed and approved by Vietnam National Cancer Hospital’s ethics committee. Written informed consent to participate in this study was provided by the participants’ legal guardian/next of kin.

## Author Contributions

DN: study design, performance of the study, data collection, statistical analysis, interpretation, and manuscript writing. DN, DL: data analysis and interpretation. DL performance of the study and data collection. DN, DL, and QL: performance of the study. All authors participated in the critical review and approval of the final manuscript.

## Conflict of Interest

The authors declare that the research was conducted in the absence of any commercial or financial relationships that could be construed as a potential conflict of interest.

## Publisher’s Note

All claims expressed in this article are solely those of the authors and do not necessarily represent those of their affiliated organizations, or those of the publisher, the editors and the reviewers. Any product that may be evaluated in this article, or claim that may be made by its manufacturer, is not guaranteed or endorsed by the publisher.
